# A vasopressin gate transforms social distress into caregiving

**DOI:** 10.1172/JCI210876

**Published:** 2026-10-01

**Authors:** Jonathan P. Fadok

**Affiliations:** 1 Department of Psychology, School of Science and Engineering, and; 2 Tulane Brain Institute, Tulane University, New Orleans, Louisiana, USA.; 3 Southeast Louisiana VA Healthcare System (SLVHCS), New Orleans, Louisiana, USA.

## Abstract

Another individual’s distress can provoke withdrawal and avoidance behaviors, yet, in social species, it can also motivate approach and caregiving behavior. In this issue, Geng et al. identified a vasopressin-sensitive neuronal circuit that converts distress signals from a cagemate into caregiving-like behavior in mice. Using fiber photometry, electrophysiological recordings, and optogenetic activation to dissect this circuit, they showed that vasopressin neurons in the paraventricular hypothalamus recruit vasopressin 1a receptor–expressing (V1aR-expressing) neurons in the medial central amygdala, which engage the ventral tegmental area to promote allogrooming and injury-directed licking. The findings link emotional state matching, prosocial action, reinforcement, and stress relief, while raising important questions about circuit specificity, sex as a biological variable, and translation.

## Shared distress creates a diversity of responses

The distress of another individual is itself a potent stimulus. It can capture attention, elevate arousal, and promote avoidance. In social species, however, the same signal can motivate approach, touch, and targeted care. Empathy is often discussed as the capacity to share or understand another’s emotional state, but shared distress alone does not necessarily lead to prosocial behavior. It must be coupled to motivational and motor systems that select an appropriate behavioral response (1–3). How the brain transforms another’s negative state into an organized action that benefits the distressed individual is a central question in social neuroscience.

Rodent models have made it possible to study this transformation in an experimental setting. Prairie voles and mice allogroom distressed partners, and mice can direct licking toward a site of injury (4–6). These behaviors should be described cautiously as caregiving-like rather than equated with the full cognitive experience of human empathy. Even so, they allow investigators to separate several processes that are difficult to disentangle in people, such as detection of social distress, emotional state matching, approach, action selection, and reinforcement. In this issue, Geng and colleagues used distress-evoked allogrooming in mice to study caregiving-like behaviors and define a subcortical pathway linking these components (7).

## A vasopressin gate for care

Geng et al. modeled distress-induced allogrooming with pairs of mice: a “demonstrator” mouse and an “observer” mouse that was already familiar to the demonstrator. The authors injected melittin, the main pain-producing component of honeybee venom, into the nape of a demonstrator mouse, producing ongoing pain at a location the animal could not easily groom. After reunion, a familiar observer rapidly investigated the demonstrator and then performed allogrooming and injury-targeted allolicking. Blocking peripheral pain in the demonstrator reduced these responses, indicating that the observer was responding to the partner’s pain state rather than the injection procedure alone. A transparent divider that preserved visual, olfactory, and auditory contact while preventing touch increased circulating corticosterone in observers. Moreover, antagonizing corticotropin-releasing factor receptor 1 reduced caregiving-like behavior, and the amount of caregiving correlated negatively with corticosterone in both members of the pair. Together, these findings support a reciprocal model in which social stress drives caregiving, and caregiving then limits stress transmission. This interpretation is consistent with evidence that reducing affective arousal can impair helping in rats (8), although corticosterone measured at the end of an interaction cannot by itself resolve the moment-to-moment internal state of either animal.

Geng et al. next identified arginine vasopressin (AVP) signaling as a molecular gate for this transformation. In the central nervous system, AVP secretion has been linked to social behavior via its actions in limbic and mesolimbic pathways, which involve the amygdala, hypothalamus, and ventral tegmental area (VTA), among other regions. c-Fos mapping revealed increased c-Fos expression in the central amygdala of observer mice, while enriched V1aR expression in the medial central amygdala (CeM) identified this subregion as a candidate mediator of caregiving-like responses. Global deletion of V1aR, or local V1aR knockdown in CeM, impaired emotional state matching, prosocial preference, and caregiving-like behavior. By contrast, oxytocin-null mice showed intact behavior. This distinction is notable because AVP and oxytocin can engage neighboring but functionally distinct populations in the central amygdala (9, 10). Fiber photometry measured increased population calcium signals from CeM V1aR neurons during the observer mouse’s initial investigation of a demonstrator mouse in pain and again at the onset of allogrooming and allolicking. Ablating or inhibiting these neurons reduced caregiving, whereas their activation evoked social approach and allogrooming even toward an uninjured cagemate. Injury-directed licking remained contingent on a partner in pain, suggesting that CeM V1aR activity provides a permissive action signal, while sensory or cortical information preserves behavioral specificity.

The upstream source of AVP secretion in the CeM was the paraventricular nucleus of the hypothalamus (PVN), a region positioned to couple social information to physiological arousal (11). Monosynaptic tracing identified PVN AVP input to CeM V1aR neurons. Disrupting PVN AVP neurons reduced caregiving, whereas optogenetic activation of these neurons or their CeM terminals initiated the behavior. Electrophysiological recordings in brain slices provided an important mechanistic link: AVP depolarized CeM V1aR neurons, stimulation of PVN AVP terminals reproduced this effect, and a V1aR antagonist blocked it. The response persisted when ionotropic glutamate receptors were blocked. This unusually complete set of anatomical, physiological, pharmacological, and behavioral experiments strongly supports direct peptidergic recruitment of the CeM.

## Reinforcing prosocial action

The circuit did not end with action initiation. CeM V1aR neurons projected to the VTA, and inhibiting their VTA terminals reduced spontaneous caregiving. Optogenetic activation of VTA terminals promoted allogrooming and allolicking, supported real-time place preference, and reduced corticosterone in observers exposed to a distressed cagemate behind a divider. These results place the VTA at the intersection of prosocial motivation, positive valence, and stress relief. The most important conceptual advance is the circuit architecture linking distinct brain areas to promote prosocial function: A negatively valenced social signal recruits PVN AVP input to a CeM gate for caregiving; CeM output engages the VTA, reinforcing an action that reduces stress in both animals (Figure 1). This feedback loop offers a biological explanation for why caregiving can recur despite being triggered by an aversive event. It also fits broader evidence that positive affect can reinforce prosocial behavior in humans (12).

## Unresolved questions

Several questions will determine how broadly this model applies. First, the VTA is not a unitary reward center; it contains molecularly and anatomically distinct dopamine, GABA, and glutamate populations with divergent outputs (13). The identity of the VTA neurons receiving CeM V1aR input, and the pathways through which they reinforce caregiving or regulate the hypothalamic-pituitary-adrenal axis, remain unknown. It will also be important to test whether motivation, motor patterning, positive valence, and stress relief are carried by the same CeM-to-VTA projection or by separable branches. Likewise, population photometry cannot determine whether single CeM V1aR neurons sequentially encode social distress and caregiving actions, or whether distinct ensembles are intermixed. Cell-resolved recordings across the full interaction could distinguish these possibilities.

Second, the pathway Geng et al. identified is best viewed as a core subcortical scaffold rather than a complete circuit for empathy. The sensory inputs that recruit PVN AVP neurons are unknown, and cortical systems likely provide context, experience, and action specificity. The anterior cingulate cortex, for example, has been implicated in selecting allogrooming versus injury-directed allolicking (6). Emotional state matching between individuals also requires further temporal resolution using simultaneous autonomic, hormonal, and neural measurements. Finally, most mechanistic experiments were performed in male mice, despite the strong dependence of AVP systems on sex and social context (11). Testing unfamiliar animals, different relationships and stressors, and both sexes will reveal whether this pathway is a dedicated caregiving circuit or a more general mechanism that converts social arousal into affiliative action.

Translation of these findings should therefore remain measured. Allogrooming in mice is not human empathy, and the intact behavior of oxytocin-null mice does not exclude oxytocin from other forms of care or from compensation during development (14). Nonetheless, the study suggests a useful organizing principle. Prosocial deficits could arise from failures to detect distress, share arousal, convert arousal into action, or experience reinforcement from helping. These are biologically distinct problems that may look similar at the behavioral level. Human studies linking amygdala responsiveness and VTA activity to empathic concern are consistent with this framework, although they do not establish circuit homology (15, 16). Geng et al. move the field beyond asking where empathy resides and toward a more tractable question: how does the brain transform another’s distress into care?

## Conflict of interest

The author has declared that no conflict of interest exists.

## Funding support

- Merit Review Award # I01 BX006434 from the United States (U.S.) Department of Veterans Affairs, Biomedical Laboratory Research and Development Service.

## Figures and Tables

**Figure 1:**
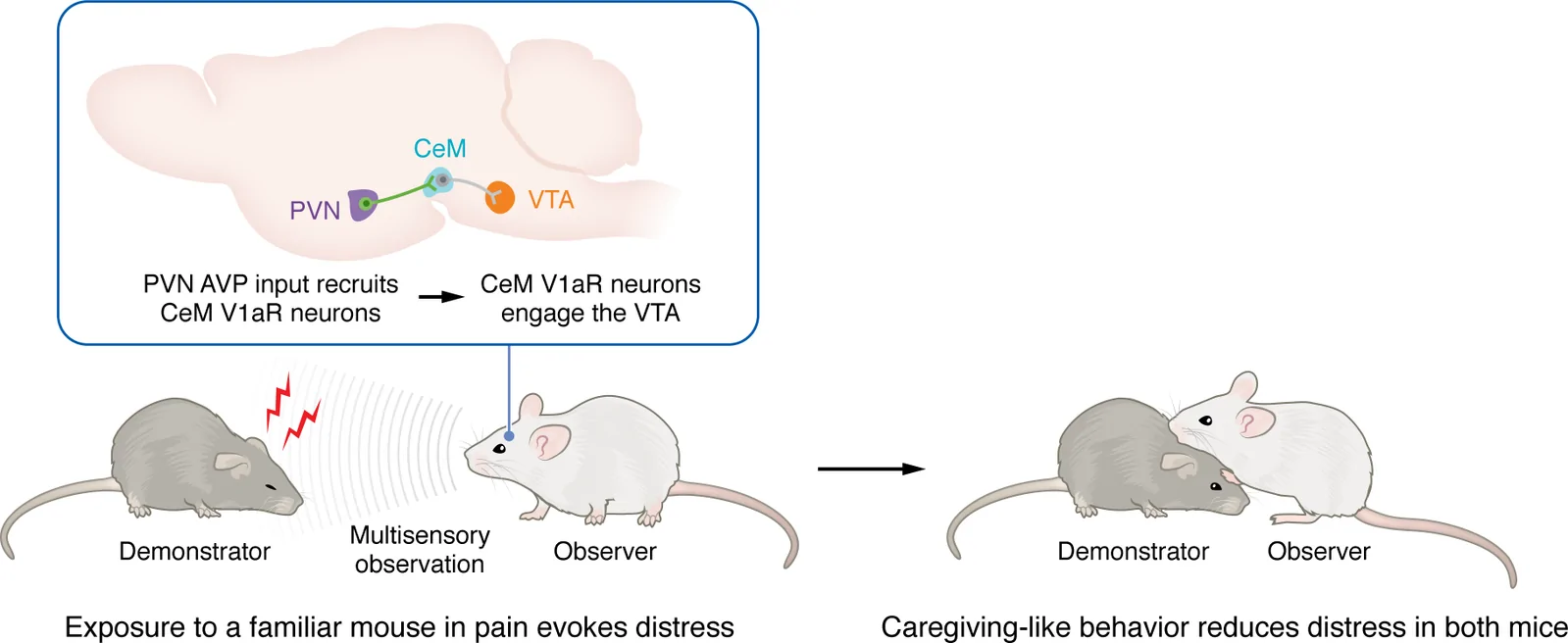
A hypothalamic-amygdala-midbrain circuit transforms shared distress into caregiving-like behavior. Geng et al.’s findings support a model wherein exposure to a familiar demonstrator in pain induces emotional state matching in an observer. Arginine vasopressin (AVP) released from paraventricular hypothalamic nucleus (PVN) terminals in the medial central amygdala (CeM) acts on vasopressin 1a receptor–expressing (V1aR-expressing) neurons. These neurons project to and engage the ventral tegmental area (VTA) to promote allogrooming and injury-targeted allolicking. VTA engagement provides positive reinforcement that may support recurrent caregiving-like behavior. Caregiving is accompanied by reduced corticosterone in both animals, consistent with reciprocal social buffering.

## Version 1 10/01/2026

Electronic publication
